# Actively station: Effects on global cognition of mature adults and healthy elderly program using eletronic games

**DOI:** 10.1590/1980-57642016dn11-020011

**Published:** 2017

**Authors:** Tiago Nascimento Ordonez, Felipe Borges, Camila Sato Kanashiro, Carolina Carneiro das Neves Santos, Samara Santos Hora, Thais Bento Lima-Silva

**Affiliations:** 1 Escola de Artes, Ciências e Humanidades da Universidade de São Paulo. Gerontologia, São Paulo SP, Brazil; 2 Faculdade de Medicina de Ribeirao Preto - Saúde em Comunidade Universidade de Sao Paulo, Ribeirao Preto SP, Brazil; 3 Grupo de Neurologia Cognitiva e do Comportamento do Departamento de Neurologia da Faculdade de Medicina da Universidade de São Paulo, São Paulo SP, Brazil

**Keywords:** elderly, cognition, cognitive stimulation, electronics equipment and games, idosos, cognição, estimulação cognitiva, equipamentos eletrônicos e jogos

## Abstract

Studies show that aging is accompanied by decline in cognitive functions but also indicate that interventions, such as training on electronic games, can enhance performance and promote maintenance of cognitive abilities in healthy older adults.

**Objective:**

To investigate the effects of an electronic game program, called Actively Station, on the performance of global cognition of adults aged over 50 years.

**Methods:**

124 mature and elderly adults enrolled in the "Actively Station" cognitive stimulation program of São Caetano do Sul City, in the State of São Paulo, participated in training for learning of electronic games. Participants were divided into two groups: training group (TG) n=102 and control group (CG) n=22. Protocol: a sociodemographic questionnaire, the Mini-Mental State Examination (MMSE), the Addenbrooke's Cognitive Examination Revised (ACE-R), the Memory Complaint Questionnaire (MAC-Q), the scale of frequency of forgetfulness, the Geriatric Depression Scale (GDS-15), the Geriatric Anxiety Inventory (GAI), the Global Satisfaction with Life Scale, and two scales on learning in the training.

**Results:**

The cognitive performance of the TG improved significantly after the program, particularly in the domains of language and memory, and there was a decrease on the anxiety index and frequency of memory complaints, when compared to the CG.

**Conclusion:**

These findings suggest that the acquisition of new knowledge and the use of new stimuli, such as electronic games, can promote improvements in cognition and mood and reduce the frequency of memory complaints.

## Abstract (pt)

Estudos mostram que o envelhecimento é acompanhado por declínio nas funções cognitivas, mas também indicam que as intervenções como o treinamento de jogos eletrônicos podem melhorar o desempenho e promover a manutenção de habilidades cognitivas em idosos saudáveis.

**Objetivo:**

Investigar os efeitos de um programa de jogos eletrônicos, denominado Estação Ativamente no desempenho da cognição global de adultos com mais de 50 anos.

**Métodos:**

124 adultos maduros e idosos inscritos no "Programa de estimulação cognitiva: Estação Ativamente" da Prefeitura Municipal de São Caetano do Sul, do estado de São Paulo. Participaram de um treinamento para aprendizagem de jogos eletrônicos. Os participantes foram divididos em dois grupos: grupo treino n=102 e grupo controle (GC) n=22. Protocolo: um questionário sociodemográfico, o Mini Exame do Estado Mental (MEEM), o Exame Cognitivo de Addenbrooke's Revisado (ACE-R), o Memory Complaint Questionnaire (MAC-Q), uma escala de freqüência de esquecimentos, a Escala de Depressão Geriátrica (GDS-15), a Escala de Ansiedade Geriátrica (GAI), a escala de satisfação geral com a vida e duas escalas sobre a aprendizagem do treinamento.

**Resultados:**

Observou-se que o desempenho cognitivo do GT melhorou significativamente após participação no programa, especificamente nos domínios de linguagem e memória, houve diminuição no índice de ansiedade e na frequência de queixas de memória, quando comparado ao GC.

**Conclusão:**

Estes achados sugerem que a aquisição de novos conhecimentos e o uso de novos estímulos como os jogos eletrônicos, podem trazer ganhos à cognição, ao humor e à diminuição da frequência de queixas de memória.

## INTRODUCTION

The shift in the demographic pyramid reflects the exponential growth of the elderly population worldwide, yet the majority of this contingent tends to feel excluded by the functional declines associated with aging that can affect vision, hearing, self-esteem, motor coordination, short-term memory, concentration and reaction abilities, among others.^1^

In general, the younger generation is confident in using technology and assimilates change easily as, since their early years, they have explored electronic toys and/or played on mobile phones. However, the older generation, born before the development of the digital world, has more difficulty accessing these technologies. In addition, some elderly individuals may lack the motor skills needed to competently use digital devices.^1-4^

Numerous studies have shown that older adults are interested and able to achieve independence in the use of computer games.^2^ These investigations also reveal that contact with technology can yield benefits such as improved social interaction and mental stimulation.^3,4^

Studies show that aging is accompanied by decline in cognitive functions but research also indicates that cognitive interventions can enhance performance and promote maintenance of cognitive abilities in healthy older adults.^5-7^ Such interventions have also been associated with functional preservation.^8-10^

Regarding electronic games use by older adults, there are a number of studies on postural control, balance, fall prevention, physical activity incentives, and functional performance.^1-3^ The positive effects of using games include improving physical function, decreasing depression, and enhancing cognition and quality of life in older adults. Improved socialization and motivation to exercise have also been reported.^1-3^ Most of the studies assessing older adults in virtual tasks involved older adults with acquired physical and cognitive diseases, while healthy older adults were only part of the control group.^3^ However, research into the use of games as a means of facilitating the use of computers and reducing loneliness of healthy older adults is scarce.^10^

Many modalities of cognitive training have been tested in both healthy and cognitively-impaired elderly.^11-15^ Shapira et al.^16^ studied a sample of Israeli elderly and found that group learning of the use of computer games and of active searching on the Internet in old age promoted a significant improvement in aspects such as depression, loneliness and self-control. The results demonstrated that Internet use can contribute to well-being and a sense of empowerment in interpersonal interactions, improving cognitive functioning and contributing to feelings of autonomy and independence.

Chen et al.^17^ performed a study comparing the sense of psychological well-being among older adults wishing to learn how to play computer games with those who did not. In the study, those elderly interested and able to achieve this goal (learning information technology for surfing the web and playing computer games) attained higher scores on the personal growth scale applied. The authors highlighted that these elderly perceived themselves as growing, developing individuals, had more goals, and a greater sense of direction in life compared to those showing no interest in learning new skills, such as use of computers and the Internet.

We investigated the effects of the stimulation program on participants' global cognition. We also examined the impact of program participation on depressive symptoms, anxiety symptoms, memory complaints and learning satisfaction in adults older than 50 years.

## METHODS

Participants. A total of 124 mature and adults older than 50 years enrolled on the "Actively Training" run by the São Caetano do Sul City Hall were recruited for the study. Participants were divided into two groups: training group (TG) and control group (CG).

The TG initially comprised 102 individuals, two of which were later excluded: one for dropping out of the activity and the other for being diagnosed with Alzheimer's Disease. The excluded subjects took part in the intervention but their data were not included in the analyses. Thus, the TG contained 102 participants, 12.87% male and 87.13% female, aged 50-89 years (M=68.43; SD=9.42). The CG comprised 22 individuals, 21.05% male and 78.95% female, aged 50-87 years (M=69.57; SD=9.08). The total sample (TG and CG) consisted of 124 mature and older adults.

The inclusion criteria adopted in this study were: being a resident of São Caetano do Sul, aged >50 years, having sufficient sensory faculties (hearing and vision) to take part in the interventions, basic motor locomotion skills, no previous diagnosis of dementia or depression, and being available to participate in the socioeducational activity centered on memory and in the computer cognitive training.

All participants of the CG and TG scored ≥25 points on the Mini-Mental Status Examination (MMSE),^18^ whereas subjects scoring below the education-adjusted cutoff scores for dementia were excluded. Participants who had no complaints of cognitive decline or history of neurologic or psychiatric illness, and not in use of drugs that could affect the central nervous system were included. None of the participants fulfilled the Diagnostic and Statistical Manual-IV^19^ criteria for depression; all scored <6 points on the Geriatric Depression Scale (GDS).^20^

**Protocol.** The sociodemographic data were collected using a questionnaire including the following variables: gender, age, schooling, marital status, family income, occupation (working and/or retired)

**Global cognition.** The Addenbrooke Cognitive Exam-Revised (ACE-R)^21,22^ consists of a brief cognitive assessment battery testing five different cognitive domains. The highest score is 100 points, distributed as follows: attention and orientation (18); memory (35); verbal fluency (14); language (28); and visuospatial abilities (5). Higher scores indicate better performance. The scores regarding each of the six domains can be computed separately and their sum gives the total ACE-R score of which 30 points are derived from the MMSE.^18^

**Memory complaints.** In order to examine memory complaints, the Memory Complaint Questionnaire (MAC-Q)^23^ was applied. Respondents are asked if their performance today is the same, better, much better, worse or much worse than it was when they were 18 years old on six memory domains. The maximum score is 35 points, and the higher the score, the greater the presence of memory complaints. A score above 25 on this instrument is considered suggestive of the presence of memory complaints.^23,24^

An adapted version of the frequency of forgetfulness (EFE) scale by McNair^25^ and Vianna-Paulo^26^ was also used. Based on this scale, participants indicated how often they forgot passwords, peoples' names, among other commonly forgotten items, selecting Never (0), Sometimes (1), Frequently (2), or Always (3). Scores range from 0 to 45 points on the scale, with higher scores denoting greater frequency of memory complaints or forgotten items. To date, no studies have established a cut-off point, for frequency of forgotten items, which would be indicative of clinically significant memory deficits.

**Screening depression and anxiety.** The Geriatric Depression Scale - GDS-15: the GDS is one of the most commonly used measures for screening depression in the elderly population. In the present study, a brief version of the instrument in Portuguese consisting of 15 questions with answers classified as yes or no, and a cut-off point of 5/6 (non-case/case), was adopted. Total score on the GDS is calculated based on the sum of the responses and indicates extent of depressed mood, with 0 being the lowest score and 15 the highest. The version used was adapted from Yesavage et al.^27^ and is considered a valid and reliable scale for use in Brazilian samples.^20,28^

The Geriatric Anxiety Inventory (GAI) was used to assess geriatric anxiety symptoms.^29,30^ The GAI consists of 20 dichotomous items and a score of 12 or higher suggests the presence of generalized anxiety disorder.

**Subjective well-being.** The Overall Satisfaction with Life (OSL) scale: a five-point scale ranging from 1 (very unsatisfied) to 5 (highly satisfied) created by Neri^31^ to measure subjective well-being indicated by satisfaction with life for three domains: health and physical capacity, mental capacity and social involvement.

**Satisfaction with training.** Upon program conclusion at six months, the participants of the Actively Station training answered a semi-structured questionnaire containing questions related to satisfaction, evaluation and attitudes regarding technological games.^4,32^ The questionnaire consisted of 21 Likert-type items, each with four response alternatives. At the end of this questionnaire, based on two open questions, the participants were asked to say what aspects of the course they most and least liked during the training.

**Description of study venue.** The study was carried out at the community center for the Third Age of São Caetano do Sul city. The city, situated in the Greater ABC- São Paulo area, is renowned nationally for its policies promoting active healthy aging and for having high population aging. According to data from the Seade Foundation, 35.8% (n 53,963) of the local population was 50 years of age or older in the months: June, July and August of 2016. In addition, the city ranks as having the highest Human Development Index in Brazil.^33^

The metropolitan human development index (MHDI) is a measure that includes the pillars of education, income and health. Among these dimensions, São Caetano do Sul has the eighth best performance for health, i.e. life expectancy number ranks among the top ten in the country (78.20 years). In order to maintain high quality of life, the city has been investing in specific services and programs for this contingent of the population since 1988. Currently, besides social programs offering paid job opportunities, the city has an Open University for the Third Age and four community centers for the Third Age, locally referred to as Integrated Centers for Health and Education of the Third Age (CISE, acronym in Portuguese).

The CISEs are social support facilities based on intersectoral partnerships that provide services in the areas of health, education, leisure, sports, culture, social welfare, legal aid, well-being and tourism to its 19,500 registered users.

**Procedures and ethical aspects.** Each of the 12 training sessions included a 45-minute educational intervention aimed at offering information about memory and aging, and at changing negative beliefs about aging, memory and health. This educational component followed a predetermined protocol. During each session, different memory subsystems were explored and aspects of memory which tend to remain stable with aging were highlighted. Special attention was given to the stability observed in immediate memory, semantic memory and implicit memory. After the educational intervention, participants were divided into small groups, led by Gerontologists, and spent 45 minutes learning how to use the electronic games equipment.

The electronic games equipment was Japanese and purchased by the sponsors of this study after visits to leisure centers for the elderly in Japan, where electronic games stations are common for health promotion and activities for prevention of cognitive decline in the population of different age groups. All the equipment used was adapted for use in Brazilian elderly people, such as: design, ergonomic adaptation, audiovisual and language of software. These changes were implemented via a pilot stage with a group of ten elderly people which took place one month before the intervention stage of the study. These adaptations were implemented with the aid of information system and computational engineering professionals. The training courses offered to the elderly in this study, as well as the manual adapted to replicate the methodology used and the guidelines for the purchase of the equipment, are available from the authors upon request by e-mail .

In all sessions, participants of the intervention trained for 15 minutes on each item of electronic equipment, starting the training with stimulation of attention and working memory (on the Taikô equipment), then episodic memory training with cards (equipment involving memory of figures that began with the presentation of 12 cards). Subsequently, subjects trained attention on the equipment with the use of the hammer. Finally, they played a game on the dance equipment, concluding participation on the program in a fun and interactive way (games described in Appendix).

All games had graded levels of difficulty. Every three consecutive encounters, participants were placed on a higher difficulty level, starting from beginner level, progressing to easy, then moderate and finally, the difficult level. Although the participants in the control group did not perform the intervention, they were placed on a waiting list, followed by the same number of sessions. They were followed by gerontologists with experience in cognitive stimulation, who monitored them in small groups of ten participants at most.

The study was conducted in compliance with International ethics standards (Declaration of Helsinki).

**Analysis of results.** The information gathered by applying the instruments was first submitted to descriptive statistical analysis. In order to describe the sample profile according to the several study variables, frequency tables of categorical variables, and descriptive statistics including measures of position and spread (mean, standard deviation, median and maximum) of continuous variables, were constructed.

The Chi-square test comparing proportion of a given response between groups was employed to determine associations for responses of the categorical variable gender between the groups.^34^ The continuous variables of interest in this study were analyzed using the Kolmogorov-Smirnov test. The test confirmed that the data required non-parametric tests. Therefore, given the nonnormal distribution of the data, the ordinal and continuous variables for the groups were compared using the Mann-Whitney test. The Wilcoxon test for related or paired samples was used to compare the before and after difference in the groups for total and domain scores on the instruments applied.

Cronbach's α coefficient was used to analyze the internal consistency reliability of the responses on the Satisfaction, Assessment and Attitudes in Relation to the Actively Station training. Alpha values ≥0.60 indicate moderate consistency and ≥0.70 high consistency.^35^ Data were keyed into the Excel Office 2010 application and subsequently analysed using the statistics software package Statistica v.7.0 (2004). The level of significance adopted for the statistical tests was 5%, i.e., p-value <0.05.

## RESULTS

A total of 124 mature and older adults took part in the study, comprising 102 participants of the Actively Station Training (Training Group-TG) and 22 control subjects (CG). The EG consisted of 88 women and 14 men, whereas the CG contained 17 women and 5 men. The means and standard deviation (SD) of age and education in the EG were 68.57 (9.53) and 8.91 (4.91) years, respectively. The CG had a mean (SD) age of 70.23 (8.25) years and education of 8.41 (4.22) years (Table 1).

**Table 1:** Socio-demographic profile of participants.

Variable		Global			Groups					p-value
					**Training**			**Control**		
		**N**	**%**		**N (102)**	**%**		**N (22)**	**%**	
Sex	Male	19	15.32		14	13.73		5	22.73	
	Female	105	84.68		88	86.27		17	77.27	0.288
Age (in years)	Mean (SD±)	68.86	9.31		68.57	9.53		70.23	8.25	
	Median	69.00			68.00			71.50		
	Minimum-maximum	50.00-89.00			50.00-89.00			52.00-84.00		0.359
Education	Elementary School (incomplete)	38	30.65		33	32.35		5	22.73	
	Elementary School (complete)	23	18.55		16	15.69		7	31.82	
	High school (incomplete)	5	4.03		4	3.92		1	4.55	
	High school (complete)	23	18.55		20	19.61		3	13.64	
	Higher education (incomplete)	11	8.87		9	8.82		2	9.09	
	Higher education (complete)	22	17.74		18	17.65		4	18.18	
	Mean (SD±)	8.82	4.78		8.91	4.91		8.41	4.22	
	Median	8.00			8.00			8.00		
	Minimum-maximum	0.00-27.00			0.00-27.00			1.00-16.00		0.893
Retired	No	27	21.77		24	23.53		3	13.64	
	Yes	96	77.42		78	76.47		19	86.36	

^a^Chi-square test. ^b^Mann-Whitney test.

The individuals in the TG and CG were matched for sociodemographic variables. At a 95% confidence level, there was no significant difference between the groups for gender, age, education or retirement status (Table 1).

Comparison of the performance of the individuals belonging to the same group TG over time revealed a significant increase in total score on the ACE-R and its subdomains: attention and orientation, verbal fluency, visuospatial ability and the Mini-Mental State Exam (MMSE). In the CG, scores differed only for language (Table 2).

**Table 2:** Comparison pre and post-test between training and control group.

Variable	Group	Time	Descriptive Statistics					
			**Mean**	**SD**	**Minimum**	**Median**	**Maximum**	**p-value**
Orientation and attention	Training	Pre-test	16.09	1.97	8.00	17.00	18.00	0.006
		Post-test	16.52	2.12	7.00	17.00	18.00	
	Control	Pre-test	15.86	2.21	8.00	16.50	18.00	0.272
		Post-test	16.13	2.00	11.00	16.00	18.00	
Memory	Training	Pre-test	19.59	4.79	4.00	20.00	26.00	0.991
		Post-test	19.80	4.72	4.00	20.00	26.00	
	Control	Pre-test	18.14	5.19	8.00	19.00	26.00	0.241
		Post-test	18.87	4.88	11.00	19.00	25.00	
Fluency	Training	Pre-test	8.97	2.78	3.00	9.00	14.00	0.047
		Post-test	9.72	2.64	4.00	10.00	14.00	
	Control	Pre-test	8.68	2.36	3.00	9.00	12.00	1.000
		Post-test	9.40	2.29	6.00	9.00	14.00	
Language	Training	Pre-test	23.10	2.95	12.00	24.00	26.00	0.005
		Post-test	24.08	2.13	15.00	25.00	26.00	
	Control	Pre-test	22.27	4.10	11.00	24.00	26.00	0.008
		Post-test	24.00	2.73	17.00	25.00	26.00	
Visuo-spatial skills	Training	Pre-test	13.79	2.08	8.00	14.00	19.00	0.019
		Post-test	14.52	1.96	6.00	15.00	16.00	
	Control	Pre-test	13.45	2.58	8.00	13.50	16.00	0.784
		Post-test	14.00	1.77	9.00	14.00	16.00	
MMSE	Training	Pre-test	26.26	2.98	11.00	27.00	30.00	0.017
		Post-test	27.07	2.73	13.00	28.00	30.00	
	Control	Pre-test	26.18	3.69	12.00	27.00	30.00	0.826
		Post-test	26.33	2.55	19.00	27.00	29.00	
ACE-R/ Total	Training	Pre-test	81.55	11.67	37.00	84.00	99.00	0.004
		Post-test	84.68	10.41	43.00	86.00	98.00	
	Control	Pre-test	78.41	12.62	44.00	81.00	94.00	0.060
		Post-test	82.40	10.47	57.00	86.00	97.00	

* Wilcoxon test for related or paired samples. ** ACE-R: Addenbrooke's Cognitive Examination-Revised. MMSE: Mini-Mental State Examination.

Comparison of the performance of the individuals belonging to the same group (TG or CG) over time revealed a significant decrease in memory-related complaints (MAC-Q) and anxiety levels in the TG (Table 3).

**Table 3:** Comparison pre and post-test between training and control groups.

Variable	Group	Time	Descriptive Statistics					
			**Mean**	**SD**	**Minimum**	**Median**	**Maximum**	**p-value**
GDS	Training	Pre-test	2.77	2.55	0.00	2.00	13.00	0.569
		Post-test	2.50	2.38	0.00	2.00	11.0	
	Control	Pre-test	2.59	2.06	0.00	2.00	7.00	1.000
		Post-test	2.40	2.56	0.00	2.00	9.00	
OSL	Training	Pre-test	3.70	0.58	1.63	3.75	5.00	0.389
		Post-test	3.70	0.90	0.00	3.88	5.0	
	Control	Pre-test	3.76	0.50	2.50	3.88	4.50	0.638
		Post test	3.88	0.63	2.50	3.88	4.88	
EFE	Training	Pre-test	9.53	4.50	1.00	9.00	22.00	0.594
		Post-test	9.17	4.13	1.00	9.00	18.0	
	Control	Pre-test	10.41	5.20	0.00	9.00	19.00	0.077
		Post-test	9.80	6.20	4.00	8.00	25.00	
MAC-Q	Training	Pre-test	25.04	3.92	12.00	26.00	35.00	<0.001
		Post-test	22.34	4.39	13.00	23.00	29.00	
	Control	Pre-test	25.68	3.68	18.00	25.00	33.00	0.263
		Post-test	26.27	3.49	20.00	27.00	34.00	
GAI	Training	Pre-test	6.96	5.37	0.00	6.00	19.00	0.011
		Post-test	6.35	5.51	0.00	5.00	19.00	
	Control	Pre-test	7.05	4.32	0.00	7.00	18.00	0.423
		Post-test	6.20	4.30	0.00	5.00	15.00	

* Wilcoxon test for related or paired samples. ** GDS: Geriatric Depression Scale (GDS). OSL: Overall Satisfaction with Life. EFE: Frequency Scale of Forgetfulness. MAC-Q: Memory Complaint Questionnaire. GAI: Geriatric Anxiety Inventory.

**Satisfaction, assessment and attitudes regarding the actively station training.** Data for Satisfaction, Assessment and Attitudes for the Actively Station Training are given in Table 4. This scale showed excellent internal consistency, as evidenced by the Cronbach coefficient of 0.873. With regard to the training participants' responses, the individuals generally rated the project positively, particularly for clarity and objectivity, doubts and queries, and machines.

**Table 4:** Satisfaction, evaluation and attitudes regarding training on games for Training Group (TG).

Variable	Subdomains	Descriptive statistics				
		**Mean**	** SD**	**Minimum**	**Median**	**Maximum**
Equipment	Ease of use	3.05	0.68	1.00	3.00	4.00
	Utility	3.44	0.55	2.00	3.00	4.00
Teachers	Clarity and Objectivity	3.64	0.48	3.00	4.00	4.00
	Participation encouragement	3.67	0.47	3.00	4.00	4.00
	View Forum Posts	3.59	0.55	2.00	4.00	4.00
Content	Ease of use	3.16	0.62	2.00	3.00	4.00
	Utility	3.45	0.58	2.00	3.00	4.00
	Usability	3.35	0.58	2.00	3.00	4.00
Intervention	Duration	3.10	0.70	2.00	3.00	4.00
	N° of participants	3.00	0.69	2.00	3.00	4.00
	Equipment	3.60	0.65	1.00	4.00	4.00
	Func. of equipment	3.47	0.60	2.00	4.00	4.00
	Facilities	3.38	0.59	2.00	3.00	4.00
Post Training	Cognition	2.90	0.65	1.00	3.00	4.00
	Strategies	2.94	0.65	1.00	3.00	4.00
	Games	3.29	0.51	2.00	3.00	4.00
	Free Online Games	3.10	0.63	1.00	3.00	4.00
	Interpersonal Relations	3.07	0.65	2.00	3.00	4.00
	Social Support	3.19	0.54	2.00	3.00	4.00
	Interest in courses	3.43	0.60	2.00	3.00	4.00
	Participation	3.26	0.62	2.00	3.00	4.00
Total Score		3.29	0.35	2.38	3.33	4.00

* Evaluation of duration, physical space, infrastructure and number of participants.

Chart 2 below shows the aspects that the training participants most liked. Four categories of aspects were derived from content analysis: I liked the monitors, the teaching approach, mixing with others and expanding my knowledge.

**Chart 2:** Responses for "what you liked most during the training".

Categories	Responses
Monitors	For the training I liked everything, especially the teachers.
Teaching Method	The didactic and practical part.
Interaction with others	Socialization, motor activities, memorization activities, cognitive activities.
Knowledge Gain	Knowledge of techniques that help us to keep our brains active.

With regard to the aspects least liked by the participants of the training, according to participant reports, one category was cited by most participants: duration of the training (Chart 3).

**Chart 3:** Responses for "what you least liked during the training".

Category	Responses
Duration of training	The duration was short. The length of the workshop, its duration should be one year. Should have more classes or a continuation of the course.

## DISCUSSION

The objective of the present study was to assess the effects of the stimulation program on participants' global cognition, depressive symptoms, anxiety symptoms, memory complaints and learning satisfaction in adults older than 50 years. In the present study, after participating in the Actively Station intervention, the individuals from the training group showed significant improvement in language performance compared to the subjects from the control group. This may suggest that the individuals in the TG performed the semantic search faster, with improvement in verbal memory and processing speed skills for verbal materials. Studies such as that by Bopp and Verhaeghen^36^ have documented maintenance of semantic and verbal memory during the normal aging process, where this may allow greater improvements when these are abilities stimulated.

The findings reported by Machado^37^ showed that after use of computers and computer games, the elderly participants felt satisfaction with their own performance, expressing feelings of inclusion in the technological and social world. In the present study, satisfaction with the teacher, the content and positive attitudes in relation to the IT training had a strong influence on participant improvements. The importance of the motivation provided throughout the learning process, and not only during the initial stages, was highlighted.

In the same vein, the study by Tomporowski^38^ revealed that, despite learning difficulties due to sensory and short-term memory decline, elderly students, when properly guided and motivated, tend to feel satisfied and supported, and consequently learn new information as effectively as younger students.

Besides these results for satisfaction and motivation, Keyes^39^ stated that critical stimulation on the intellectual task being undertaken by older adults can aid the learning process. This critical stimulation is a way of promoting the adaptation and flexibility of elderly with regard to the teaching model put in practice in an intervention for the third age.^40^ In the present study, the use of historical facts about IT and the Internet and the focus on information of common interest such as health and aging during the classes, probably promoted this stimulation.

The preparation of printed materials for the course also aided participant learning. The feedback on actions, prior preparation of instructions and clear outlining of the main objective of the tasks to be learned proved fundamental activities of the researcher in teaching elderly about Internet use.^40^

Missotten et al.^41^ and Ready et al.^42^ reported that cognitive decline negatively impacts QoL. Therefore, it follows that improvements in cognitive can positively influence self-rating of life. The results found on the EDEP were congruent with the findings of Irigaray et al. (2011) who reported significant improvement in the TG on the domains Environment, Personal Growth, Selfacceptance and Create after training episodic memory, attention and executive functions. However, unlike the study by Irigaray et al.,^43^ the present investigation employed scores for factors identified in a previous study on this scale in Brazil.^44^

Nevertheless, no single formula is universally valid for all older adults and therefore, when devising a digital inclusion training program, it is important to take into account who the elderly are, where they come from, and what they expect from the service. Knowledge on all relevant aspects of the aging process is required to structure an effective program reflecting the reality of the participants involved.

For the majority of the older adults, learning computer games was a special achievement. Many reported during the interventions that they now play games with their children at family get-togethers. Curiosity to learn new things led to the perception by the elderly that there are no limits to realizing desires, dreams or overcoming obstacles. Based on the results of the present study, it can be concluded that the use of technology by the older adults contributed to mental health and to achieving good quality of life during old age. The interaction during and outside the training also helped reduce social isolation and loneliness.

Critical stimulation during teaching, clear and objective language, recognition of students' knowledge, and planning of actions by the researcher were fundamental for efficacy of learning. The inclusion of the elderly in the world of IT and learning, the development of new methods, and discussions on achieving this, appear to be goal of common interest to both public and private educational institutions.^4^ At the University of São Paulo, there are examples where these goals have been sought since implementation of the Open University of the Third Age in 1993.^45^

A previous Brazilian training study that included healthy older adults, failed to find changes in metamemory variables (memory complaints), such as memory self-efficacy and knowledge about memory, after training,^46^ in contrast to studies carried out with healthy American elderly,^47,48^ where subjective memory complaints were common in the elderly population.^49^ These differences may be associated with the instruments used or with the training methodology. Indeed, changes in subjective memory may require the use of an intervention protocol which includes goal-setting and feedback information, matched to cognitive tasks organized in increasing level of difficulty.^48^ In other words, a psycho-educational protocol with information on memory and aging, such as the one reported in the current training protocol, seems to be insufficient to generate changes in perceptions about cognition.

Overall, the Actively Station project has produced promising results. It has provided a space for interaction of young and older adults and promoted a rich array of communication through digital and technological interactivity. The project has given both these age groups the opportunity to discover and build new meanings, particularly with respect to the social roles which they can and wish to engage in throughout their aging process. Elderly individuals, who are expected to merely convey experiences and knowledge, have put themselves into a situation where they know little or nothing about a specific context or sphere, and can experience the challenge of experimenting the new and a sense of inexperience in which they assume the role of a student.

This study provides important contributions, because it shows that interventions with electronic games can stimulate and promote better cognitive performance in Brazilian adults and elderly without dementia or depression. It should be highlighted that the gerontological literature emphasizes the importance of cognitive preservation by promoting the maintenance of autonomy, independence and lowering risks of hospitalization and institutionalization in the elderly. The program could be replicated in other Brazilian cities as a health promotion measure and public policy in Gerontology. This study has some limitations, including the fact that the participants were recruited from the setting of a health and education center for the elderly, where results reported were representative of active older adults. The effect of the cognitive training on satisfaction with learning can be increased. Additionally, the participants of the TG were not compared against a group receiving another type of intervention or to an active CG, a comparison which could have allowed the effect of interacting in a group to be identified. Also, the distribution of participants across the groups was not fully randomized and may have introduced some bias in favor of the TG. Future studies should replicate the method of this study with a larger number of sessions and fewer participants to further elucidate the relationship among learning, cognitive performance, satisfaction, motivation, attitudes and beliefs with regard to technology.

## Figures and Tables

**Chart 1:** Services offered to visitors of the Integrated Health and Education Centers of the city of São Caetano do Sul.

Department	Services offered
Cheers	Outpatient care in nursing, medicine, geriatrics, physiotherapy, psychology, hydrotherapy, nutrition and dentistry.
Education	Language and literacy courses.
Recreation	Ecumenical events, festivals and dances.
Sports	Gymnastics, bodybuilding, stretching, adapted sports, Pilates, walking and water aerobics.
Culture	Performing arts, plastic arts, visual arts, literature and music workshops.
Social assistance	Crafts workshops, patchwork, macrame, needlework, mosaic, rag doll, embroidery, wicker, carton.
Legal Assistance	Legal assistance once a week
Welfare	Hairdressing, manicure and pedicure
Tourism	Sociocultural Tourism Program of the 3rd Age - tours to museums, parks, local fairs, cinemas and trips to the countryside and coast of São Paulo and other states.

Source: Collection of the city of São Caetano do Sul, 2016, http://www.saocaetanodosul.sp.gov.br/

## References

[1] Anderson F, Annet M, Boschof WF (2010). Lean on Wii: physical rehabilitation with virtual reality Wii
peripherals. Studies in Health Technology and Informatics.

[2] Sposito LAC, Portela ER, Bueno EFP, Carvalho de WRG, Silva da FF, De Souza RA (2013). Experiência de treinamento com Nintendo Wii sobre a
funcionalidade, equilíbrio e qualidade de vida de
idosas. Motriz rev. educ. fis.(impr).

[3] Sales MB (2002). Desenvolvimento de um checklist para a avaliação de
acessibilidade da Web para usuários idosos.

[4] Ordonez TN, Yassuda MS, Cachioni M (2011). Elderly online: effects of a digital inclusion program in
cognitive performance. Arch Gerontol Geriatr.

[5] Ball K, Berch DB, Helmers KF, Jobe JB, Leveck MD, Marsiske M (2002). Effects of cognitive training interventions with olders adults: A
randomized controlled trial. J Am Med Assoc.

[6] Irigaray TQ, Gomes I, Schneider RH (2012). Efeitos de um treino de Atenção, Memória e
Funções Executivas na Cognição de Idosos
Saudáveis. Psicol Refl Crít.

[7] Lima-Silva TB, Yassuda MS (2012). Treino cognitivo aliado á ação
psicoeducativa em idosos hipertensos: efeitos na
cognição. Psicol Refl Crít.

[8] Rebok GW, Ball K, Guey LT, Jones RN, Kim HY, King JW (2014). Ten-Year Effects of the Advanced Cognitive Training for
Independent and Vital Elderly Cognitive Training Trial on Cognition and
Everyday Functioning in Older Adults. J Am Geriatr Soci.

[9] Willis SL, Tennstedt SL, Marsiske M (2006). Long-term effects of cognitive training on everyday functional
outcomes in older adults. J Am Med Assoc.

[10] Bagwell DK, West RL (2008). Assessing compliance: active versus inactive trainees in a memory
intervention. Clin Interv Aging.

[11] Carvalho FCR, Neri AL, Yassuda MS (2010). Treino de memória episódica com ênfase em
categorização para idosos sem demência e
depressão. Psicol Refl Crít.

[12] Kawashima R (2013). Mental Exercises for Cognitive Function: Clinical
Evidence. J Prev Med Public Health.

[13] Silva HS, Yassuda MS (2009). Memory training for older adults with low education: mental
images versus categorization. Educ Gerontol.

[14] Ordonez TN, Cachioni M (2009). Universidade Aberta à Terceira idade: a experiência
da Escola de Artes, Ciências e Humanidades. Rev Bras Ciênc Envelhec Hum - RBCEH.

[15] Lima-Silva TB, Oliveira ACV, Paulo DLV, Malagutti MP, Danzini VMP, Yassuda MS (2011). Treino cognitivo para idosos baseado em estratégias de
categorização e cálculos semelhantes a tarefas do
cotidiano. Rev Bras Geriatr Gerontol.

[16] Shapira N, Barak A, Gal I (2007). Promoting older adults' well-being through Internet training and
use. J Aging Mental Health.

[17] Chen Y, Persson A (2002). Internet use among young and older adults: Relation to
psychological well-being. Educ Gerontol.

[18] Brucki SMD, Nitrini R, Caramelli P, Bertolucci PHF, Okamoto IH (2003). Sugestões Para o Uso do Mini-Exame do Estado
Mental. Arq Neuropsiquiatr.

[19] American Psychiatric Association (1994). Diagnostic and statistical manual of mental disorders.

[20] Paradela EMP, Lourenço RA, Veras RP (2005). Validação da Escala de Depressão
Geriátrica em um ambulatório geral. Rev Saúde Pública.

[21] Mioshi E, Dawson K, Mitchell J, Arnold R, Hodges JR (2006). The Addenbrooke's Cognitive Examination Revised (ACE-R): a brief
cognitive test battery for dementia screening. Int J Geriatr Psych.

[22] Carvalho VA, Barbosa MT, Caramelli P (2010). Brazilian Version of the Addenbrooke Cognitive
Examination-revised in the Diagnosis of Mild Alzheimer
Disease. Cogn Behav Neurol.

[23] Croock TH, Feher EP, Larrabee GJ (1992). Researsh and Reviews, Assessment of Memory Complaint in
Age-Associated Memory Impairment: The MAC-Q. Int Psychogeriatrics.

[24] Mattos P, Lino V, Rizo L, Alfano A, Araújo C, Raggio R (2003). Memory complaints and test performance in healthy elderly
persons. Arq Neuropsiquiatr.

[25] McNair M, Kahn R, Crook T, Ferris S, Bartus R (1983). Self-assessment of cognitive deficits. Assessment in Geriatric Psychopharmacology.

[26] Paulo DLV, Yassuda MS (2010). The relation between memory complaints in the elderly and
education, cognitive performance, and symptoms of depression and
anxiety. Rev Psiquiatr Clín.

[27] Yesavage JA, Brink TL, Rose TL, Lum O, Huang V, Adey M, Leirer VO Development and validation of a depression screening scale: A
preliminary report. J Psychiatr Res..

[28] Almeida OP, Almeida SA (1999). Reliability of the Brazilian version of the ++abbreviated form of
Geriatric Depression Scale (GDS) short form. Arq Neuropsiquiatr.

[29] Pachana NA, Byrne GJ, Siddle H, Koloski N, Harley E, Arnold E (2007). Development and validation of the Geriatric Anxiety
Inventory. Int Psychogeriatr.

[30] Massena PN, de Araújo NB, Pachana N (2014). Validation of the Brazilian Portuguese Version of Geriatric
Anxiety Inventory - GAI-BR. Int Psychogeriatr.

[31] Neri AL (1998). Escala para a avaliação de satisfação na
vida referenciada a domínios.

[32] Villar F (2003). Personas mayores y ordenadores: valoración de una
experiencia de formación. Rev Esp Geriatr Gerontol.

[33] Human Development Index in Brazil data reference in 2013.

[34] Vieira S (1980). Introdução à Bioestatística.

[35] Cronbach LJ (1951). Coefficient alpha and the internal structure of
tests. Psychometrika.

[36] Bopp KL, Verhaeghen P (2007). Age-related differences in control processes in verbal and
visuo-spatial working memory: Storage, transformation, supervision, and
coordination. J Gerontol Psychol Sci.

[37] Machado LR (2007). Metas motivacionais de idosos em inclusão digital.

[38] Tomporowskizz PD (2003). Effects of acute bouts of exercise on cognition. Acta Psychol.

[39] Keyes CLM, Shmotkin D, Ryff CD (2002). Optimizing well-being: The empirical encounter of two
traditions. J Personal Soc Psychol.

[40] Aleven V, Stahl E, Schworm S, Fischer F, Raven W (2003). Help seeking and help design in interactive learning
environments. Review Educ Res.

[41] Missotten P, Squelard G, Ylieff M, Di Notte D, Paquay L, De Lepeleire J (2008). Relationship between quality of life and cognitive decline in
dementia. Dement Geriatr Cogn Disord.

[42] Ready RE, Ott BR (2003). Quality of life measures for dementia. Health Quality Life Outcomes,.

[43] Irigaray TQ, Schneider RH, Gomes I (2011). Efeitos de um treino cognitivo na Qualidade de vida e no
Bem-estar Psicológico de Idosos. Psicol Refl Crít.

[44] Queroz NC, Neri AL (2005). Bem-estar psicológico e inteligência emocional
entre homens e mulheres na meia-idade e na velhie. Psicol Refl Crít.

[45] Ordonez TN, Cachioni M (2009). Universidade Aberta à Terceira idade: a experiência
da Escola de Artes, Ciências e Humanidades. Rev Bras de Ciênc Envelhec Hum- RBCEH.

[46] Yassuda MS, Batistoni SST, Fortes AG, Neri AL (2006). Cognitive training for healthy older adults: benefits and
mechanisms of gain. Psicol Refl Crít.

[47] West RL, Hastings EC (2011). The relative success of a self-help and a group-based memory
training program for older adults. Psychol Aging,.

[48] West RL, Bagwell DK, Dark-Freudeman A (2008). Self-Efficacy and Memory Aging: The Impact of a Memory
Intervention Based on Self-Efficacy. Aging Neuropsychol Cogn.

[49] Brigola AG, Manzini CSS, Oliveira GBS, Otaviani AC, Sako MP, Vale FAC (2015). Subjective memory complaints associated with depression and
cognitive impairment in the elderly: A systematic review. Dement. Neuropsychol.

